# Stunted girl: A heartbreaking case report of underdiagnosed and untreated posterior ankyloglossia

**DOI:** 10.1016/j.ijscr.2024.109648

**Published:** 2024-04-20

**Authors:** Robertus Arian Datusanantyo, Simplicia Maria Anggrahini, Arif Tri Prasetyo

**Affiliations:** a Department of Surgery, Prof. Dr. W. Z. Johannes General Hospital/Faculty of Medicine and Veterinary Medicine, Universitas Nusa Cendana, Kupang, Indonesia; b Department of Pediatrics, Prof. Dr. W. Z. Johannes General Hospital/Faculty of Medicine and Veterinary Medicine, Universitas Nusa Cendana, Kupang, Indonesia; c Division of Plastic Reconstructive and Aesthetic Surgery, Department of Surgery, Faculty of Medicine, Universitas Padjadjaran, Bandung, Indonesia

**Keywords:** Stunted, Ankyloglossia, Speech disorder, Eating disorder, Tongue tie, Case report

## Abstract

**Introduction:**

Plastic surgeons can help to eliminate stunting by surgically treating children born with congenital craniofacial anomalies such as tongue-tie, or ankyloglossia. Releasing ankyloglossia can help to support breastfeeding and the later development of orofacial anatomy and physiology. Failure to do so can lead to growth and development difficulties in children. We report a heartbreaking case of a stunted 8 year-old female with underdiagnosed and untreated ankyloglossia.

**Presentation of case:**

The patient was consulted with a short stature, speech disorder, and swallowing disorder. History taking and physical examination led to a diagnosis of type 4 (posterior) ankyloglossia. The Hazelbaker Assessment Tool for Lingual Frenulum Function mandated a frenotomy. Under general anesthesia, frenotomy was performed surgically, and significant tongue mobility was gained.

**Discussion:**

This case alerted both surgeon and pediatrician that collaboration is a must to intervene in such a specific congenital anomalies. Posterior (type 4) ankyloglossia may cause difficulties in tongue mobility which can lead to difficulties in breastfeeding and swallowing, speech disorders, and malocclusion. Posterior ankyloglossia is not only the most severe form of ankyloglossia, but also the most difficult to diagnose.

**Conclusion:**

In the absence of social and environmental factors, posterior (type 4) ankyloglossia was the single most responsible factor in this growth and development delay in the girl. Timely diagnosis and treatment could have prevented such a stunted condition.

## Highlights

• Plastic surgeon may contribute to growth and development of children by diagnosing and treating ankyloglossia.
• Posterior ankyloglossia is rare and difficult to diagnose.
• Failure to treat ankyloglossia may result in short stature, speech disorder, and poor daily performance.

## 1 Introduction

Indonesia, particularly in the Lesser Sunda Islands, is still burdened with stunted children [1]. While poor linear growth or stunting (less than −2 of height-for-age Z score) [2] should be defined in the first one thousand days of age, stunted children beyond this age are seen in daily practice. Stunted children result from the complex interaction of multiple factors, both individually and at the household and community levels [[1], [2], [3]]. These factors are not necessarily pathological and may be related to household size, education level, maternal health status, and antenatal care.

However, children with poor nutritional intake may end up stunted. Swallowing difficulty or oropharyngeal dysphagia (OPD), prevents infants and children from optimal growth and development due to poor nutritional intake. The oral stage of OPD can be caused by tongue-tie, or ankyloglossia [4]. Ankyloglossia is a short lingual frenulum that limits tongue mobility, thus impairing tongue function, including swallowing [5].

Plastic surgeons are commonly consulted for ankyloglossia, especially when it is difficult to diagnose and treat. Different classifications and treatments of ankyloglossia are available but not widely accepted [6]. However, ankyloglossia is commonly classified based on free tongue length or elasticity of the frenulum, and the decision to surgical intervention is based on function and appearance [5]. Several researches support releasing ankyloglossia to improve breastfeeding, and failure to do so may lead to maldevelopment of orofacial musculatures [7].

We report a case of stunted girl whose ankyloglossia was underdiagnosed and was not treated properly. This case is particularly unique because the late diagnosis and treatment have led the patient to a stunted growth and underdeveloped physical and social skill. The patient was referred to our hospital, a general referral hospital with subspecialty and surgical service. This case is reported in line with the SCARE criteria [8].

## 2 Presentation of case

A little girl was referred to the pediatric and plastic surgery outpatient clinic with a chief complaint of speech disorder, drooling, and swallowing disorder. The patient was 8 years and 3 months old, with short stature (116 cm; −2 SD = 116.3 cm) and a weight of 18 kg (−2 SD = 19.1 kg). This made the patient slightly overweight, based on a body mass index of 20.1 (+2 SD = 20.8). The patient was born at 2400 g and had completed vaccination schedule as per the Indonesian health authorities' program.

Previously, the patient has been taken by the parent to visit several health care facilities to seek assessment regarding the difficulty in swallowing and speech. The patient has never been diagnosed with ankyloglossia, and some physical therapy was programmed but the parent didn't comply to all advice and treatment due to some social and domestic difficulties.

**Fig. 1:**
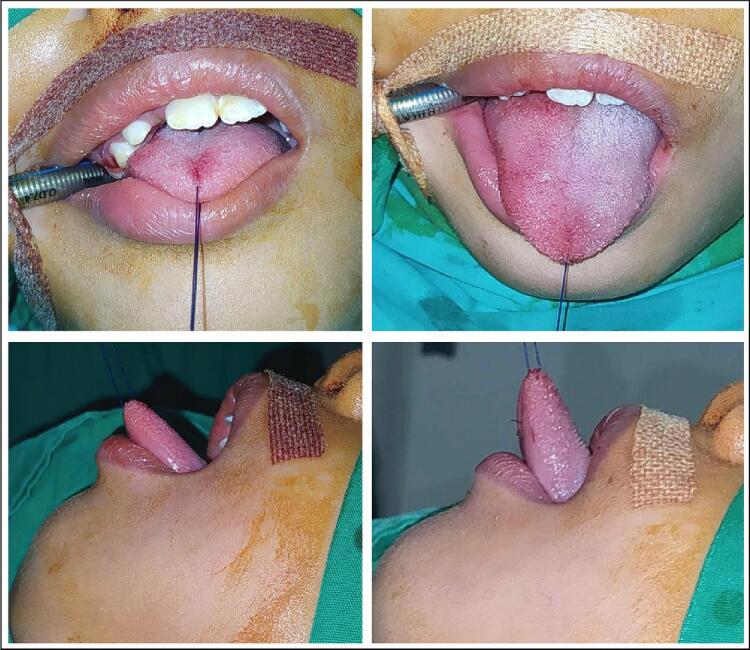
Pre- (left) and post- (right) operative photograph, notice the extension of tongue mobility.

The patient spoke very little but maintained eye contact and used hand and facial gestures to communicate. She was cooperative during all physical examinations. Physical examination confirmed the inelasticity of the lingual frenulum, which attached from the posterior tip to below the alveolar ridge. The tip of the tongue could not extend beyond her lower lip (Fig. 1). When lifted, the tip of her tongue remained at the alveolar ridge. When asked to drink or eat, the patient showed difficulty in swallowing and excess water and saliva drooled from the corners of her mouth.

The Hazelbaker Assessment Tool for Lingual Frenulum Function (HATLFF) appearance score was 7 and functional score was 10, indicating that frenotomy was necessary. The diagnosis of type 4 or posterior ankyloglossia was made, and frenotomy was performed under general anesthesia (Fig. 1).

**Fig. 2:**
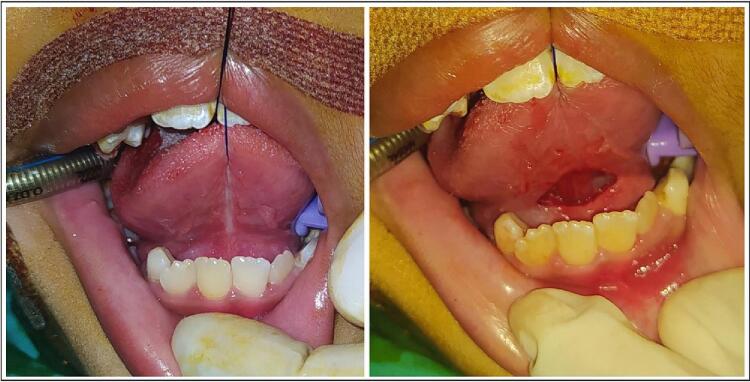
Lingual frenulum in posterior ankyloglossia: short, thick, fibrous cord-like, obscured by mucosal curtain.

**Fig. 3:**
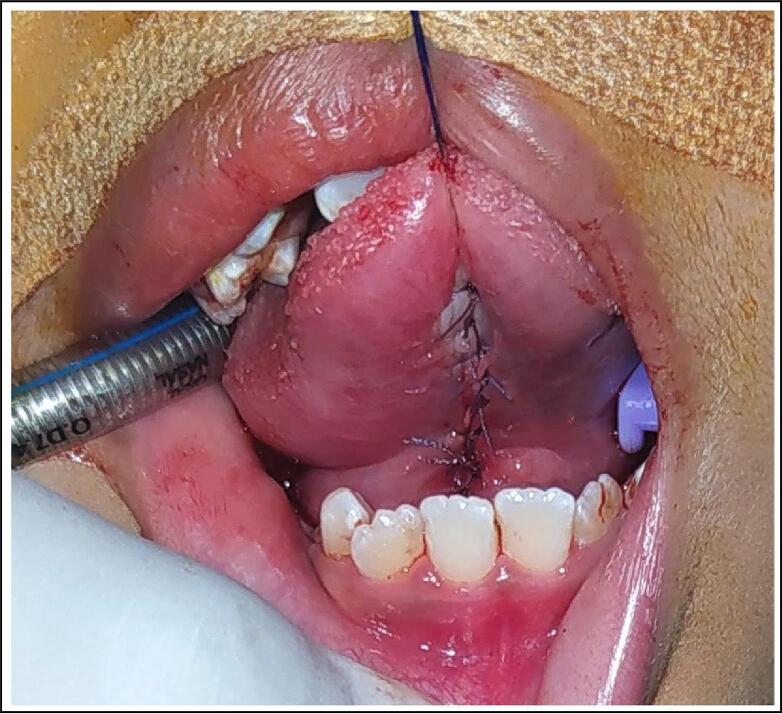
Horizontal-to-vertical frenuloplasty with absorbable suture.

Under general anesthesia, a horizontal incision was made in the mucosa to identify the thick fibrous band underneath (Fig. 2). Using both blunt and sharp dissection, the anterior tongue was released and improved mobility was gained. After hemostasis, the diamond-shaped mucosal defect was closed in a horizontal-to-vertical frenuloplasty fashion using absorbable sutures (Fig. 3).

Tongue mobility was improved, and the patient was transferred to a rehabilitation specialist to improve swallowing and speech. The patient returned to daily routine as a student of school for children with special needs. The parents observed no significant changes during the patient's routine physical and learning activity. During follow-up visits, we learned that the parents had failed to comply with the therapy schedule. Therefore, little to no functional improvement was observed on speech and swallowing post-operatively.

## 3 Discussion

Childhood stunting is the best indicator of inequalities that affect overall child well-being [9]. While stunting, by definition, is diagnosed in the first one thousand day of life, poor environmental conditions may falter children's growth beyond this milestone. The patient presented in this case was stunted. However, this condition was unlikely the result of environmental and social conditions. The child parents are both educated and have steady career. They live in urban area with plenty of access to healthcare. Thus, individual pathological condition is more likely to be responsible.

It is obvious during history and physical examination that the patient had been in continuous OPD due to ankyloglossia. This event led a series of consequences that resulted in her short stature and poor ability to perform daily task for a child her age. Segal and colleagues reported that ankyloglossia is associated with difficulties in breastfeeding, especially difficulty latching, nipple pain, and eventually risk of breastfeeding cessation [10]. A prevalence of ankyloglossia has been reported to be 5 %, but this may vary depending on the tools used in the assessment [11].

Ankyloglossia is also associated to speech disorder and malocclusion. A review by Suter and Bornstein described various mechanical limitation of the tongue in adults due to ankyloglossia, including difficulty in articulating specific consonants [6]. Ankyloglossia, although evidence is limited, may also influence the growth and development of the stomatognathic system and lead to malocclusion [6,7].

The simplest classification was proposed by Kotlow who measured tongue mobility using a dental instrument [12]. This method classifies ankyloglossia into clinically acceptable tongue mobility and four other class of ankyloglossia (Table 1). Walker and colleagues associated Kotlow's classification to breastfeeding difficulties and clearly suggested that maternal experience in breastfeeding should be an indicator of breastfeeding difficulty, as tongue mobility is associated with nipple pain [13].

**Table 1:** Ankyloglossia according to Kotlow [12].

Class	Range of free tongue
Acceptable	Normal range of >16 mm
Class I	Mild ankyloglossia, 12–16 mm
Class II	Moderate ankyloglossia, 8–11 mm
Class III	Severe ankyloglossia, 3–7 mm
Class IV	Complete ankyloglossia, <3 mm

Coryllos and colleagues proposed another classification of ankyloglossia based on how close to the tip of the tongue the leading edge of the frenulum is attached [14]. This classification divides ankyloglossia into four types (Table 2). Ankyloglossia types 3 and 4 are called the posterior ankyloglossia and their uncharacteristic appearance may make them difficult to recognize during physical examination.

**Table 2:** Types of ankyloglossia according to Coryllos and colleagues [14].

Type	Frenulum attachment
Type 1	Frenulum attached to tip of the tongue and anterior to alveolar ridge in the lower lip sulcus
Type 2	Frenulum attached 2–4 mm posterior to the tip of the tongue and just behind the alveolar ridge
Type 3	Frenulum attached to the mid-tongue and the middle of floor mouth, usually tighter and less elastic
Type 4	Frenulum is against the back of the tongue, thick, shiny, and very inelastic

While it was unfortunate for the patient to be underdiagnosed, posterior ankyloglossia itself is commonly missed when using attachment-only score [15]. In posterior ankyloglossia, the lingual frenulum is not prominent in inspection, but was short, thick or fibrous cord-like when examined using grooved director or manual palpation [16]. Chu and Bloom dramatically described it as “short, thick, fibrous cord posterior to the ventral tongue mucosa, obscured by mucosal curtain (Fig. 2)” [17].

The HATLFF is not a tool to diagnose ankyloglossia (Table 3). Instead, it quantifies functional and appearance items, and provides a recommendation for whether a frenotomy is necessary [18]. We perform this assessment on the patient and it showed impaired function and the appearance score was below 8. Both scores indicated that a frenotomy to be performed.

**Table 3:** Hazelbaker assessment tool for lingual frenulum function [18].

	2	1	0
Appearance items			
Appearance of tongue when lifted	Round or square	Slight cleft in tip apparent	Heart-shaped
Elasticity of frenulum	Very elastic (excellent)	Moderately elastic	Little or no elasticity
Length of lingual frenulum when tongue lifted	>1 cm or embedded in tongue	1 cm	<1 cm
Attachment of lingual frenulum to tongue	Posterior to tip	At tip	Notched tip
Attachment of lingual frenulum to inferior alveolar ridge	Attached to floor of mouth or well below ridge	Attached just below ridge	Attached at ridge

Function items			
Lateralization	Complete	Body of tongue but not tongue tip	None
Lift of tongue	Tip to mid-mouth	Only edges to mid-mouth	Tip stays at alveolar ridge or rises to mid-mouth only with jaw closure
Extension of tongue	Tip over lower lip	Tip over lower gum only	Neither of above or anterior to mid-tongue humps
Spread of anterior tongue	Complete	Moderate or partial	Little or none
Cupping	Entire edge, firm cup	Side edges only, moderate cup	Poor or no cup
Peristalsis	Complete, anterior to posterior (originates at tip)	Partial: originating posterior to tip	None or reverse peristalsis
Snapback	None	Periodic	Frequent or with each suck

14: Perfect score, regardless of appearance.

11: Acceptable if appearance score >10.

<11: Impaired function, frenotomy is considered if management fails. Frenotomy is necessary if appearance score is <8.

Significant gain was seen immediately after the frenotomy (Fig. 1). However, functional improvement was observed post-operatively. The parents were satisfied with the tongue mobility. Although the parents were informed and a transfer plan to a rehabilitation specialist was discussed, we were unable to observe any functional improvement after one year of follow up. The parent didn't comply with the rehabilitation schedule and the patient refused to physical treatment several times.

Unfortunately, the strength of this case is that we confirmed the collective failure that has resulted in a significant loss of this patient's potential as a daughter, child, and future community member. This is not only the devastating consequences of underdiagnosed and untreated posterior ankyloglossia, but also the collective failure to meet this patient's basic needs. While anterior ankyloglossia is easier to diagnose and accounts for 94 % of ankyloglossia [16], the minority posterior ankyloglossia is more challenging to diagnose. However, the patient had always had difficulty swallowing and later developed speech disorders. Both of these problems should have alerted the parents and healthcare professionals. Surgeons, in particular, should participate by considering posterior ankyloglossia when performing oral examination.

However, the limitation of this report is that despite anatomical gain in tongue mobility after the surgery, we failed to document significant functional improvement, especially in swallowing and speech. The parent gave written consent to this report and a clearance was granted by the hospital administrator to report and publish the case.

## 4 Conclusion

The absence of other social and environmental factors confirms that posterior (type 4) ankyloglossia was the single most responsible factor in this patient's growth and development disorder. This case implied that in clinical practice, surgeons and pediatricians should collaborate to ensure timely diagnosis and treatment of posterior ankyloglossia. Posterior ankyloglossia is not only rare and difficult to diagnose, but also leads to compromised growth. Timely diagnosis and treatment could have prevented such poor growth and development.

## Consent

Written informed consent was obtained from the patient's parents for publication and any accompanying images. A copy of the written consent is available for review by the Editor-in-Chief of this journal on request.

## Ethical approval

Approval for this case report was granted by the Prof. Dr. W. Z. Johannes General Hospital Director under an approval letter number: 445/2197/RSUD3.1.

## Funding

This research did not receive any specific funding.

## Author contribution

Robertus Arian Datusanantyo: Conceptualization; Data curation; Formal analysis; Methodology; Validation/Interpretation; Writing - original draft; and Writing - review & editing.

Simplicia Maria Anggrahini: Conceptualization; Data curation; Formal analysis; Methodology; Validation/Interpretation; Writing - original draft; and Writing - review & editing.

Arif Tri Prasetyo: Conceptualization; Data curation; Formal analysis; Methodology; Validation/Interpretation; Writing - original draft; and Writing - review & editing.

## Guarantor

Robertus Arian Datusanantyo.

Simplicia Maria Anggrahini.

Arif Tri Prasetyo.

## Research registration number

This study does not require registration.

## Conflict of interest statement

The authors declare no conflicts of interest related to this study.
